# The internal mammary artery perforator flap as a reliable option for thoracic osteoradionecrosis reconstruction: a case report

**DOI:** 10.1097/RC9.0000000000000496

**Published:** 2026-04-29

**Authors:** Amina Oyuntogos, Pharel Njessi, Olivier Camuzard, Elise Lupon

**Affiliations:** a Department of Plastic and Reconstructive Surgery, Institut Universitaire Locomoteur et du Sport, Pasteur 2 Hospital, University Côte d’Azur, Nice, France; b CNRS LP2M, Université Côte d’Azur, Nice, France

**Keywords:** breast carcinoma, case report, complex soft tissue defects, IMAP flap, perforator flap, radionecrosis

## Abstract

**Introduction and importance:**

One of the common late complications following radiotherapy is radionecrosis of the surrounding tissue, which can progress to infection, chronic wound, or even sepsis. Surgical reconstruction remains the standard treatment for this condition, as conservative management is ineffective once necrosis and bone exposure occur. We report a case of pedicled internal mammary artery perforator (IMAP) flap to cover a defect of right supraclavicular and clavicular defect created by cutaneous and osteoradionecrosis.

**Case presentation:**

24 years after mastectomy and radiotherapy for right breast carcinoma, lesion with moderate hypermetabolic activity was found on PET CT. After inconclusive biopsy findings, the sternocostoclavicular joint lesion was fully resected. The wound was closed directly, and final histopathology confirmed no evidence of tumor proliferation. Postoperative complication of delayed wound healing, and local infection occurred requiring daily wound care for 7 months. Surgical reconstruction with IMAP flap was performed, followed by complete recovery of chronic clavicular wound and no reduced mobility of upper extremities.

**Clinical discussion:**

This case illustrates the reconstructive challenges posed by late radiation-induced tissue injury and highlights the perforator flap’s role as an anatomically favorable option when prior surgeries, fibrosis, or vascular compromise limit conventional alternatives.

**Conclusion:**

The successful outcome demonstrates that the pedicled IMAP flap provided durable, functional coverage for complex post-radiation cervicothoracic defects in patients with few viable reconstructive options. This case underscores its utility in managing delayed osteoradionecrosis with chronic wound.

## Introduction

Radionecrosis is a common late complication of radiotherapy, occurring in up to 30% of cases, with a higher incidence in females. This complication directly correlates with the radiotherapy prescription dose and frequency, and the tumor size. The diagnosis is based on clinical symptoms, pathological findings and radiographic imaging^[^1^]^. Cutaneous radionecrosis and osteoradionecrosis may remain asymptomatic in the early stages, sometimes being detected incidentally on imaging years after treatment. When symptomatic, patients typically present with delayed wound healing, erythema, pain, tenderness, swelling, bone exposure or fistula formation, which can lead to infection, abscess, or sepsis^[^2^]^.

HIGHLIGHTS

- Chronic osteoradionecrosis can present decades after breast cancer radiotherapy and mimic recurrent malignancy on imaging.
- Pedicled internal mammary artery perforator (IMAP) flap provided reliable, well vascularized coverage in a heavily irradiated cervicothoracic area.
- The IMAP flap achieved complete wound healing, preserved shoulder function, and durable results at 20-month follow-up.

The standard management involves surgical debridement of all the necrotic tissue, followed by reconstruction using skin graft or local or free flaps. Reconstruction of radionecrotic defects of the anterior thoracic wall has historically relied on muscular or musculocutaneous flaps such as latissimus dorsi (LD), transverse rectus abdominis myocutaneous, and pectoralis major flap, as well as fasciocutaneous flaps such as deltopectoral (DP) flap^[^2–4^]^. Although these remain reliable options, they are associated with notable drawbacks, including greater donor site morbidity, bulkiness, limited arc of rotation, and less favorable aesthetic outcomes.

Perforator flaps have transformed thoracic wall reconstruction by enabling transfer of well-vascularized tissue while minimizing donor site morbidity. They offer superior pliability, reduced functional impairment, and improved cosmetic results compared with traditional muscular flaps. Among these, the internal mammary artery perforator (IMAP) flap has gained increasing popularity. First introduced by Yu in 2006 as a perforator-based thinner modification of the traditional DP flap, it offers excellent vascular reliability and pliability, with an ideal arc of rotation for cervicothoracic reconstruction^[^5–7^]^. The internal mammary artery, a branch of the subclavian artery, runs approximately 1 cm lateral to the sternum, giving rise to perforators through the intercostal spaces, which can be utilized as the vascular pedicle for a pedicled or free IMAP flap, allowing for versatile rotation and reliable perfusion even in previously irradiated regions.

The present case describes IMAP flap reconstruction for chronic clavicular osteoradionecrosis occurring more than two decades after breast cancer radiotherapy, complicated by persistent non-healing wound despite months of conservative management and prior extensive thoracic surgery. This case required both diagnostic clarification and tailored multidisciplinary reconstructive planning in an irradiated, anatomically altered field. This case is presented in accordance with the SCARE guidelines^[^8^]^.

## Case presentation

A 74-year-old female patient was referred to us for management of chronic right clavicular osteoradionecrosis following a biopsy. She presented with a 7-month non-healing wound requiring daily wound care. Her medical history includes right breast carcinoma treated in 1999 with mastectomy reconstructed using a LD perforator flap, right axillary lymph node dissection, and radiotherapy of right mastectomy area. She is a former smoker (20 packs per year) and has a surgical history of total hysterectomy, bilateral ovariectomy, omentectomy, as well as right foot cutaneous carcinoma treated with excision and skin grafting in 2023. Her comorbidities include ischemic stroke with right-sided brain ischemic lesion, atrial fibrillation treated with catheter ablation in 2022, anticoagulation with apixaban for thromboembolic prevention, hypertension, and dyslipidemia.

Twenty-four years after her initial oncologic treatment, a retractile infiltrate of the right apex extending to the subclavicular region with osteolysis of the first rib and clavicle was discovered on imaging. PET-CT demonstrated moderately hypermetabolic activity. She was admitted to the thoracic surgery department for biopsy to rule out possible sarcoma. Histopathology showed pluricellular fibro-inflammatory, with dystrophic and locally necrotic changes. Some aspects suggested the possibility of a low-grade tumor with cartilaginous differentiation, although this could not be formally confirmed based on the samples. Next generation sequencing (45 gene panel) showed no mutations. Clinically, following biopsy, the patient developed localized redness suggestive of radiodermatitis.

**Figure 1.**
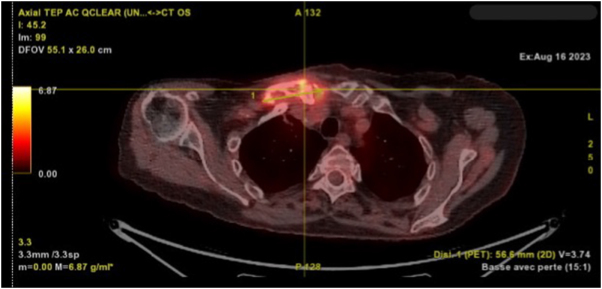
PET CT showing lytic osteoarticular lesion at the right sternocostoclavicular junction, measuring approximately 56 mm. *Printed with permission; copyrights retained by Elise Lupon, MD, PhD.*

A follow-up PET CT (Fig. 1) performed 14 weeks after the initial PET CT and one month after the biopsy showed progression of a lytic osteoarticular lesion at the right sternocostoclavicular junction, measuring approximately 56 mm along its longest transverse axis (compared to 48 mm previously). The hypermetabolic focus of lytic tissue and osteoarticular involvement persisted at the right sternocostoclavicular junction.

**Figure 2.**
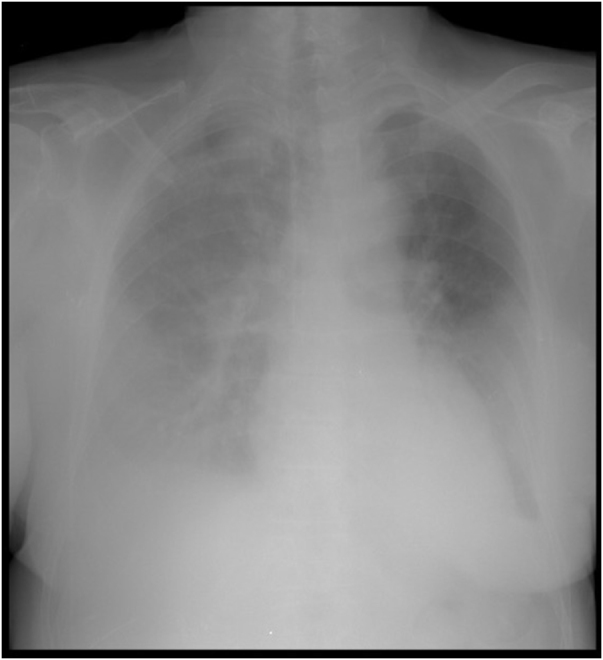
Postoperative X-ray after the excision of the right sternocostoclavicular joint including half of the manubrium, a segment of the clavicle, and the first rib via direct approach was performed. *Printed with permission; copyrights retained by Elise Lupon, MD, PhD.*

After multidisciplinary thoracic discussion, surgical excision was recommended. Excision of the right sternocostoclavicular joint including half of the manubrium, a segment of the clavicle, and the first rib via direct approach was performed (Fig. 2). The second histopathologic analysis revealed chronic fibrous changes consistent with post-radiation effects, with superimposed acute necrotic inflammatory foci, and no evidence of tumor proliferation.

Postoperatively, the wound healing was significantly delayed. The patient developed wound dehiscence and surrounding erythema. Bacterial culture of the serous wound fluid grew *Staphylococcus aureus*, and the patient was treated with intravenous cefazolin and negative pressure wound therapy.

**Figure 3.**
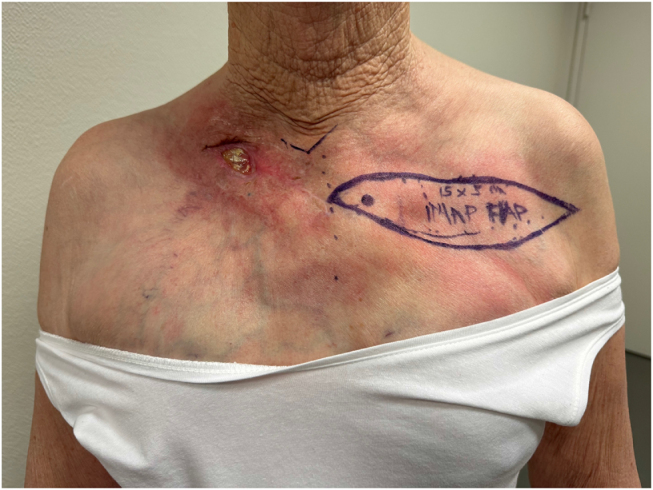
Preoperative planning of IMAP flap on the contralateral, non-irradiated side, with perforator localization using acoustic Doppler. *Printed with permission; copyrights retained by Elise Lupon, MD, PhD.*

After 7 months of daily wound care, the patient presented with exposed right clavicular osteophytes with an open wound of 4-cm with localized redness (Fig. 3). She was admitted for surgical reconstruction following the decision of the multidisciplinary reconstructive team.

**Figure 4.**
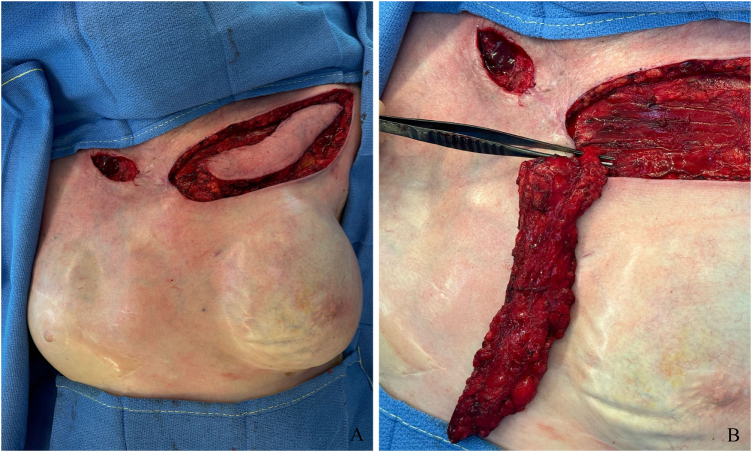
(A) Per-operative view of dissected internal mammary artery perforator flap. (B) Dissected pedicle arising from the second intercostal space of the internal mammary artery and vein. *Printed with permission; copyrights retained by Elise Lupon, MD, PhD.*

**Figure 5.**
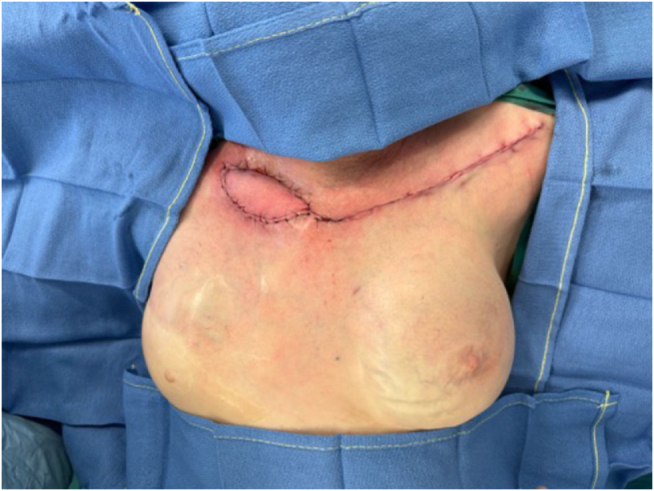
Immediate postoperative view with donor area closed directly, and 15 × 5 cm IMAP flap rotated 180° degrees. *Printed with permission; copyrights retained by Elise Lupon, MD, PhD.*

An IMAP flap was done to surgically cover the exposed right clavicle. The flap was designed measuring 15 × 5 cm, to cover the distal part of the affected tissue. Acoustic Doppler was used to locate the perforator, and the direct closure of the donor site was confirmed by pinch test (Fig. 3). The distal portion of the flap was incised, followed by suprafascial dissection of its distal half with monopolar dissector. The medial half was dissected subfascially toward the parasternal region using dissecting scissors. After the flap perforator was identified and isolated (Fig. 4), it was rotated 180° degrees to reach the defect. The necrotic and ulcerated tissues were excised. The donor site was closed without tension, and the flap was then contoured to match the recipient soft-tissue defect and secured with tension-free Skoog sutures (Fig. 5).

**Figure 6.**
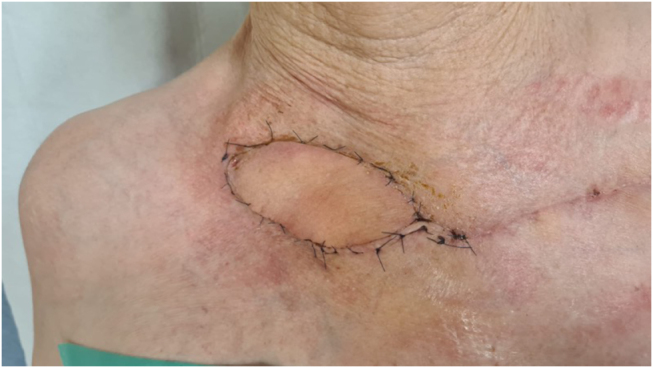
Postoperative follow-up 10 days after surgery. *Printed with permission; copyrights retained by Elise Lupon, MD, PhD.*

**Figure 7.**
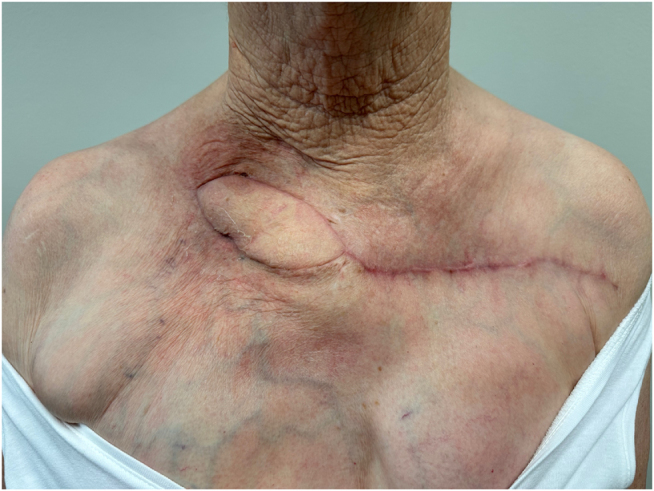
Postoperative follow-up 5 weeks after surgery. *Printed with permission; copyrights retained by Elise Lupon, MD, PhD.*

**Figure 8.**
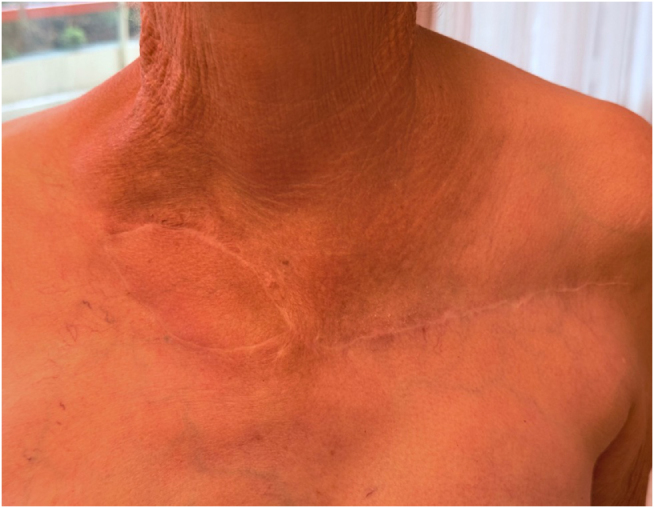
Postoperative follow-up 20 months fully healed, showing a completely viable flap with full wound closure. *Printed with permission; copyrights retained by Elise Lupon, MD, PhD.*

The flap was monitored every 3 hours for the first 24 hours, with assessment of color and capillary refill time. The patient was discharged from the hospital on POD 1 with daily dressing and monitoring of flap. Postoperative histopathology of the resected tissues showed ulceration and subacute and chronic fibro-inflammatory changes with no evidence of malignancy. At 2 weeks post-op, the patient no longer required dressing, the flap was healed completely, and the sutures were removed. There were no immediate postoperative complications (Fig. 6) and no recurrence at 5 months (Fig. 7). At 20 months post-op (Fig. 8), the flap was stable with no sign of recurred osteonecrosis or chronic wound. The patient reported resolution of chronic pain and had no restriction of upper extremity range of motion.

## Discussion

Soft tissue radionecrosis and osteoradionecrosis represent serious late complications of radiotherapy that may occur years or even decades after treatment. The pathophysiology involves progressive endarteritis obliterans, tissue hypoxia, fibrosis, and impaired cellular repair, all of which lead to chronic ulceration and poor wound healing. Reconstruction is often challenging due to radiation-induced vascular damage, fibrosis of surrounding tissues, and risk of infection, which compromise both local tissue quality and recipient vessels.

The current gold standard for management includes radical excision of all necrotic and infected tissue, followed by reconstruct using well vascularized flaps. Perforator flaps are particularly advantageous in irradiated fields because they provide reliable blood supply, thin and pliable coverage, and minimal donor site morbidity while preserving underlying muscle and function^[^3,9^]^.

In this case, free flap options such as superficial circumflex iliac perforator flap (SCIP) were excluded due to the patient’s history of smoking, post-radiation vascular uncertainty, and postoperative fibrosis, all of which increase microvascular risk. A LD flap can be used for anterior chest wall defect^[^2,4,10,11^]^ but was not feasible as it had previously been used for breast reconstruction after mastectomy, and the posterior brachial artery flap was contraindicated as a pedicled flap due to prior axillary lymph node dissection. Consequently, the IMAP flap from the contralateral side was selected as the optimal reconstructive surgical option. The IMAP flap, first described by Yu in 2006^[^5^]^, offers a reliable vascular pedicle, adequate arc of rotation, and thin, pliable tissue, making it particularly suitable for clavicular and supraclavicular defects^[^12,13^]^. Compared with DP flap, the IMAP flap provides better contour, shorter operative time, and reduced donor site morbidity. One of the principal advantages of the IMAP flap is its functional preservation. Unlike musculocutaneous flaps such as the LD or pectoralis major flaps, the IMAP flap does not compromise shoulder or chest wall mobility and maintains donor site integrity^[^12^]^. This is particularly important for elderly or previously irradiated patients in whom postoperative recovery and functional outcomes are key considerations.

Alternative strategies such as artificial dermal substitutes^[^14^]^ or skin grafting are not suitable for these defects, as they cannot provide reliable coverage when bone is exposed or when deep fibrosis is present. In such cases, only durable vascularized tissue can meet the reconstructive requirements.

This case underscores the importance of early multidisciplinary collaboration, particularly timely referral to a plastic surgeon in the context of osteoradionecrosis, to optimize outcomes in complex post-radiation wounds^[^15^]^. Early involvement can significantly reduce healing time and associated healthcare cost, as illustrated here by a patient who underwent daily wound dressings for 7 months before being referred to a plastic surgeon. This procedure can be performed on an outpatient basis by an experienced surgeon and is associated with rapid healing^[^16^]^.

The key lesson from this case is that, in patients with limited local options and compromised vascularity, the pedicled IMAP flap provides a safe, reliable, and versatile reconstructive solution for cervicothoracic defects following osteoradionecrosis. Its use should be considered early in the reconstructive management when conventional or free flap options are unavailable or contraindicated.

## Conclusions

In this case, the pedicled IMAP flap provided a reliable and versatile option, offering thin pliable tissue coverage with a dependable blood supply from outside the irradiated zone. This technique proved effective when conventional or free flaps were contraindicated. This case underlines the importance of individualized reconstructive planning in irradiated fields and presents a reliable, original technique for complex soft tissue reconstruction. It highlights the advantages of the pedicled IMAP flap as safe, adaptable, and functionally and aesthetically favorable option for post-radiation defects.

The work has been reported in line with the SCARE criteria 2025.

## Research registration unique identifying number (UIN)

1. Name of the registry: IORG0012275 - iULS-University Institute for Locomotion and Sport 2. Unique identifying number or registration ID: IRB00014528_2025_49 3. Hyperlink to your specific registration (must be publicly accessible and will be checked): not applicable as it is not the first-in-man case report.
